# Supplementary material for: Development of a novel risk score for diagnosing urinary tract infections: Integrating Sysmex UF-5000i urine fluorescence flow cytometry with urinalysis
Source: PLoS One. 2025 May 14;20(5):e0323664. doi: 10.1371/journal.pone.0323664 (PMC12077719; doi:10.1371/journal.pone.0323664)
Supplement: S1 Table — (DOCX) [file pone.0323664.s005.docx]

**S Table. Detailed definition of variables obtained from urine flow cytometry**

| **Variable** | **Description** | **Normal value** |
| --- | --- | --- |
| Red blood cell | Red blood cells deliver oxygen to the body’s tissues by travelling through the circulatory system. They are round, smooth and have a red tinge | < 23/µL |
| White blood cell | Cell of immune system reacted to inflammation and infection | < 25/µL |
| White blood cell | The aggregation of individual white blood cell into larger clusters | < 23/µL |
| Hyaline cast | Most common types of casts in urine. They are cylindrical and appear almost transparent. Hyaline casts can be found in urine due to dehydration, fever or vigorous exercise. | < 1/µl |
| Non-hyaline cast | Contain inclusions. They develop when particles – such as red blood cells or tubular epithelial cells – are present during the solidification process. Particles adhere to the fibrillar protein network and get surrounded by it. These casts appear in urine when pathological processes take place in the kidney. Examples: Granular casts, cellular casts, waxy casts. | < 1/µl |
| Squamous epithelial cell | Squamous epithelial cells are 20-100 µm in diameter, of multi-edged or rounded shape with a central or slightly off-center nucleus, depending on their superficial, intermediate or deep layer position within the epithelium. Squa.EC form the urothelium of the urethra, vulva and reproductive system. The presence of Squa.EC in urine can indicate urethritis or injury through lithiasis or catheterization. In many cases, it can be considered as a contamination. | < 31/µL |
| Non-squamous epithelial cell | Non SEC cover the epithelium of the urethra, the prostate gland and uterine cervix. Non SEC can indicate urethritis and mechanical injury but can also be a side effect of menstruation. |  |
| Transitional epithelial cell | Transitional epithelial cells are 15-150 µm in diameter, of polygonal, angular shape with a central or slightly off-center nucleus, depending on their superficial, intermediate or deep layer position within the epithelium. Tran.EC form the multi-layered urothelium, covering the renal pelvis, kidney calyx, ureter, bladder and urethra. The presence of Tran.EC in urine can point to inflammation, malignancy, or injury through lithiasis or catheterization. | < 1/µL |
| Renal tubular epithelial cell | Renal tubular epithelial cells are 10-30 µm in diameter and appear in various shapes with an eccentric nucleus. RTECs comprise the monolayer epithelium, lining the proximal renal tubule, the loop of Henle, the distal renal tubule, the collecting ducts and the renal papilla. RTECs can indicate kidney damage or upper urinary tract infections in context of bacteriuria. | <1/µL |
| Bacterial cell | Bacteria |  |
| Yeast-like-cell | Yeast cells are smooth, colorless and usually egg-shaped. They have birefringent walls, come in different sizes and often show budding. The most common type of yeast found in urine is *Candida albicans*. Yeast cells in urine can come from either skin-related or vaginal contaminations or can indicate mycotic urinary tract infections. | < 1/µl |
| UTI flag | Urinary tract infection detected by the machine |  |
