# Supplementary material for: Development of a novel risk score for diagnosing urinary tract infections: Integrating Sysmex UF-5000i urine fluorescence flow cytometry with urinalysis
Source: PLoS One. 2025 May 14;20(5):e0323664. doi: 10.1371/journal.pone.0323664 (PMC12077719; doi:10.1371/journal.pone.0323664)
Supplement: Attachment — Submitted filename: Response to reviewer.docx [file pone.0323664.s008.docx]

**RESPONSE TO REVIEWERS**

**Reviewer #2**

**Comment #1:** Thank you for addressing all of the comments in your revised manuscript. I have no additional suggestions at this time. However, I would recommend a final proofreading to further enhance the readability of the paper and to correct any remaining grammatical errors.

**Response to comment #1:** We agreed and thank the reviewer for this comment. We proofread the manuscript and revised to improve the readability.

**Reviewer #3**

**Comment #1:** The detailed clarification of the inclusion criteria now effectively addresses concerns about selection bias, clearly delineating the initial diagnosis process. This enhances the manuscript's transparency and robustness. The explanation provided about the selection of retrospective cases, including detailed matching criteria, significantly strengthens the alignment of these cases with the study’s objectives, ensuring that the validation cohort supports the research findings effectively.

**Response to comment #1:** We thank the reviewer for his comments and suggestion.

**Journal Requirements:**

Please review your reference list to ensure that it is complete and correct. If you have cited papers that have been retracted, please include the rationale for doing so in the manuscript text, or remove these references and replace them with relevant current references. Any changes to the reference list should be mentioned in the rebuttal letter that accompanies your revised manuscript. If you need to cite a retracted article, indicate the article’s retracted status in the References list and also include a citation and full reference for the retraction notice.

**Response:** We have checked the reference to ensure they are complete and correct.
